# Prognosis value of Hypoxia-inducible factor-1α expression in patients with bone and soft tissue sarcoma: a meta-analysis

**DOI:** 10.1186/s40064-016-3064-x

**Published:** 2016-08-19

**Authors:** Yongjiang Li, Wenbiao Zhang, Shuangjiang Li, Chongqi Tu

**Affiliations:** 1 Department of Oncology, West China Hospital, Sichuan University, Chengdu, People’s Republic of China; 2 Department of Orthopedics, West China Hospital, Sichuan University, 37 Guoxuexiang, Chengdu, 610041 Sichuan Province People’s Republic of China

**Keywords:** Bone and soft tissue sarcoma, HIF-1α, Prognosis value, Meta-analysis

## Abstract

The prognostic significance of Hypoxia-inducible factor-1α (HIF-1α) in patients with bone and soft tissue sarcoma remains controversial. To investigate the impact of its expression on survival outcomes, we performed a meta-analysis. Comprehensive literature searches were conducted in PubMed, Web of Science, Embase and Cochrane Library. A total of 16 studies published from 2006 to 2015 were included. We found that expression of HIF-1α was significantly associated with higher rate of metastasis (RR 3.21, 95 % CI 2.12–4.84, P < 0.001), poorer overall survival (HR 2.05, 95 % CI 1.51–2.77, P < 0.001) and poorer disease-free survival (HR 2.05, 95 % CI 1.55–2.70, P < 0.001). In addition, when subgroup analysis was conducted according to histology type, the significant correlations to poor overall survival and disease-free survival were also observed in patients with osteosarcoma, chondrosarcoma and soft tissue sarcoma. Publication bias was not found and sensitivity analysis showed the results were stable. In conclusion, HIF-1α expression might be an effective predicative factor of poor prognosis for bone and soft tissue sarcoma.

## Background

Sarcomas are a heterogeneous group of mesenchymal malignant tumors that can be divided into two general categories: primary bone sarcoma and soft tissue sarcoma (Skubitz and D’Adamo 2007). Primary bone sarcomas mainly include osteosarcoma, Ewing’s sarcoma, chondrosarcoma; soft tissue sarcomas mainly include synovial sarcoma, leiomyosarcoma, liposarcoma and angiosarcoma. With the emergence of effective chemotherapy regimens and the development of surgical techniques, the survival rate of sarcoma patients increased (Hwang et al. 2014). However, metastasis still occurs in 20–55 % of these patients, and it remains the main cause of death (Nakamura et al. 2009; Tsukushi et al. 2014). Efforts in the last 20 years including the changes of the chemotherapy drugs, the doses and the administration schemes did not significantly improve prognosis (Luetke et al. 2014). Advanced treatment methods are urgently needed. There is no doubt that effective prognostic factors are important for researchers and clinicians to select reasonable treatment methods for sarcoma patients (Wang et al. 2015).

Hypoxia-inducible factor-1 (HIF-1) plays a central role in cellular response to hypoxia, which is a heterodimer composing of an oxygen-liable α subunit and a constitutively expressed β subunit. In normoxic environment, HIF-1α is rapidly ubiquitinated and degradated by von Hippel–Lindau tumor-suppressor protein (Epstein et al. 2001; Jaakkola et al. 2001). In contrast, under hypoxia environment, the degradation process is suppressed and HIF-1α translocates from the cell plasma to the nucleus, where it could regulate the expression of more than 60 genes involved in crucial aspects of tumor biology (Semenza 2001; Kimura et al. 2001). Through this way, tumor cells could activate adaptive responses to match metabolic demands with oxygen supply, and survive under intratumoral hypoxia microenvironments. Overall, HIF-α expression could contribute to tumor progression in the way of sustaining energy metabolism, maintaining biosynthesis and promoting tumor cell invasion and migration.

It has been confirmed by a number of studies that expression of HIF-1α is correlated with poor prognosis in various cancers, including gastric, esophageal and lung cancers (Zhang et al. 2013; Ping et al. 2014; Wang et al. 2014). However, the prognostic role of HIF-1α expression in bone and soft tissue sarcoma has not reached a consensus since inconsistent results were reported in previous studies (Zhao et al. 2015; Kim et al. 2015; Hu et al. 2015; Guo et al. 2014; Smeland et al. 2012; Chen et al. 2012a, b, 2011; Zeng et al. 2010; Huang et al. 2010; Boeuf et al. 2010; Hoffmann et al. 2009; Mizobuchi et al. 2008; Kubo et al. 2008; Chen et al. 2008; Yang et al. 2007; Shintani et al. 2006). To date, there has been no comprehensive meta-analysis to clarify its prognostic role in sarcoma. Therefore, we conducted the current meta-analysis to combine published studies and to comprehensively assess the prognostic significance of HIF-1α expression in bone and soft tissue sarcoma.

## Methods

We conducted comprehensive electronic literature searches in PubMed, Web of Science, Embase and Cochrane Library with no restriction to language and date of publication. The last search was conducted on July 17, 2015. The search terms were as follows: (“HIF-1” OR “Hypoxia Inducible Factor-1”) AND (“sarcoma” OR “soft tissue sarcoma” OR “bone sarcoma” OR “osteosarcoma” OR “chondrosarcoma” OR “Ewing sarcoma” OR “leiomyosarcoma” OR “angiosarcoma” OR “histiocytoma” OR “liposarcoma” OR “rhabdomyosarcoma” OR “synoviosarcoma”). In addition, reference list of identified articles were traced by Google Scholar for potential studies.

Studies were eligible for inclusion if they met the following criteria: (1) included patients with pathologically confirmed bone and soft tissue sarcoma; (2) investigated the association between HIF-1α expression and the outcomes of sarcoma patients; (3) provided information on metastasis, disease-free survival or overall survival; (4) were in language of English or Chinese. The following studies were excluded: (1) reported overlapping patients; (2) non-human research; (3) reviews, letters and articles from conferences; (4) with insufficient information. When articles recruiting overlapping patients were identified, the most recent published article was included in the meta-analysis. The literatures were evaluated independently by two authors (YJ Li and WB Zhang) for eligibility. Any disagreement was discussed and adjudicated by corresponding author (CQ Tu).

### Data extraction and quality assessment

Data of interest was extracted independently by two authors (YJ Li and SJ Li). The required data included: (1) basic information of each publication including first author, year of publication, study period, follow-up duration and study design; (2) data of patient and tumor including patient source, number, age, percentage of positive HIF-1α expression and histology type of tumor; (3) outcome measures including overall survival, disease-free survival, Kaplan–Meier curves and metastasis; and (4) other variables including the methods of quantitative HIF-1α measurements and definition of positivity (the cut-off value).

Each included article’s quality was evaluated using Newcastle–Ottawa Scale (NOS) (www.ohri.ca/programs/clinical_epidemiology/oxford.asp). Based on the quality of each study in selection, comparability and exposure, a score up to 9 points was appointed. Articles with 6 or more of the NOS scores were deemed as high-quality and were included in the meta-analysis.

### Statistical analysis

To assess the prognostic significance of HIF-1α expression, we calculated the pooled hazard ratio (HR) or relative risk (RR) with its corresponding 95 % confidence interval (CI). If the HRs or RRs were given explicitly in the publications, we used the original data. If the data were not given explicitly, we calculated the HRs or RRs with 95 % CIs from outcome data available in the articles or from Kaplan–Meier curves through methods reported by Tierney et al. (2007), Xu et al. (2013), Zhuang and Wei (2014) and Kubo et al. (2015).

Heterogeneity was evaluated using Chi squared test and I^2^ statistic. If P > 0.1 and I^2^ < 50 %, the heterogeneity was not considered as significant. Otherwise, the heterogeneity was not significant. Both fixed-model and random effect model were conducted to calculate the overall estimate. Publication bias was evaluated by Egger’s test and Begg’s test. If P > 0.05 and the funnel plot was visually symmetry, it was not considered as significant. In addition, sensitivity analysis was conducted to evaluate the stability of the results by omitting individual study sequentially. All statistical analyses were conducted using STATA version 12.0 (Stata Corp., College Station, TX).

## Results

### Searches results and study characteristics

**Fig. 1:**
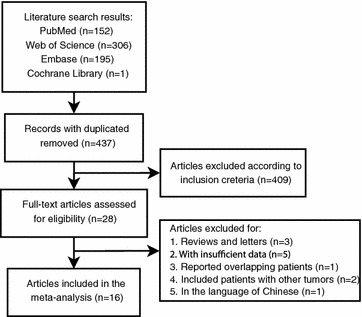
Flow diagram of study selection

A total of 437 articles were identified in the initial searches after duplicated removed. After initial screening and full-text viewing, 420 articles were removed because they do not meet our inclusion criteria. Particularly, we identified two articles recruiting overlapping patients in the full-text viewing process. After discussion, the earlier published article was excluded (Chen et al. 2012a, b). One study in Chinese language was also excluded, for its unfamiliarity for non-Chinese speakers. Eventually, 16 articles published from 2006 to 2015 were included in the current meta-analysis (Fig. 1) (Zhao et al. 2015; Kim et al. 2015; Hu et al. 2015; Guo et al. 2014; Smeland et al. 2012; Chen et al. 2008, 2012a, b, 2011; Zeng et al. 2010; Huang et al. 2010; Boeuf et al. 2010; Hoffmann et al. 2009; Mizobuchi et al. 2008; Kubo et al. 2008; Yang et al. 2007; Shintani et al. 2006). The NOS scores of the included studies are counted and shown in Table 1. All the studies have 6 or more of the NOS scores.

**Table 1:** Characteristics of eligible studies included in the meta-analysis

Study	Patient source	Study duration	Follow-up duration (range), months	Method	Antibody source for IHC	Dilution of antibody	Defination of positivity	Histology type	Mean age (range), years	Number of patients	HIF + (%)	Study design		NOS score
Zhao et al. (2015)	China	2002–2012	NR (3–107)	IHC	Novus	1:100	>25 %	OS	NR	88	56.8	Re	M	7
Kim et al. (2015)	Korea	1998–2007	38 (2–187)	IHC	Abcam	1:1000	>50 %	STS	57 (1–82)	55	54.5	Re	S	8
Hu et al. (2015)	China	2006–2011	36	IHC	Proteintech	1:200	SS	OS	NR	50	58.0	Pro	M	9
Guo et al. (2014)	China	2003–2007	NR	IHC	Abcam	1:200	>10 %	OS	NR	98	79.9	Re	S	7
Smeland et al. (2012)	Norway and Russia	1973–2006	37.6 (0.1–391.7)	IHC	Novus	1: 3500	SS	STS	60 (0–91)	200	61.5	Re	M	8
Chen et al. (2011)	China	NR	34 (4–98)	IHC	Abcam	1:50	>10 %	CS	47.9 (18–72)	34	58.8	Re	S	8
Chen et al. 2012a, b	China	2000–2009	29 (6–100)	IHC	Santa	1:100	SS	OS	18.5 (11–72)	49	55.1	Pro	S	9
Zeng et al. (2010)	China	NR	NR	IHC	NR	1:100	SS	OS	18.6 (7–49)	45	55.6	Re	S	7
Huang et al. (2010)	USA	1987–2006	27.5 (0.7–229)	IHC	Labvision	NR	>10 %	STS	64 (21–85)	39	41.0	Re	S	7
Boeuf et al. (2010)	Germany	NR	116.3	IHC	BD	1:1250	SS	CS	NR	65	33.8	Re	M	8
Hoffmann et al. (2009)	Germany	NR	NR	RT-RCP	–	–	NR	STS	57 (16–85)	45	64.4	Re	S	7
Mizobuchi et al. (2008)	USA	NR	NR	IHC	Novus	1:100	SS	OS	21 (7–38)	48	37.5	Re	S	7
Kubo et al. (2008)	Japan	1986–2001	78 (1–192)	IHC	Novus	1:1000	>10 %	CS	42 (15–71)	20	40.0	Re	S	8
Chen et al. (2008)	China	NR	NR	IHC	NeoMarkers	1:70	SS	OS	NR (3–63)	25	68.0	Re	S	6
Yang et al. (2007)	China	NR	50 (13–86)	IHC	Santa	1:50	SS	OS	19 (5–56)	39	43.6	Re	S	8
Shintani et al. (2006)	Japan	NR	39 (5–181)	IHC	Novus	1:1000	SS	STS	63 (12–85)	42	52.4	Re	S	7

*NR* not reported, *IHC* immunohistochemistry, *RT*-*PCR* reverse transcription-polymerase chain reaction, *SS* score system combining intensity and percentage, *OS* osteosarcoma, *STS* soft tissue sarcoma, *CS* chondrosarcoma, *Re* retrospective, *Pro* prospective, *M* multi-center, *S* single center, *NOS* Newcastle–Ottawa Scale

Baseline characteristics of the included 16 studies are tabulated and shown in Table 1. Briefly, all the 16 literatures were in English. Two studies were prospectively designed and 14 studies were retrospectively designed. Study sample sizes ranged from 20 to 200, and a total of 942 sarcoma patients were included. The rate of HIF-1α expression ranged from 33.8 to 79.9 % and 531 patients had positive HIF-1α expression.

### Quantitative data synthesis

**Fig. 2:**
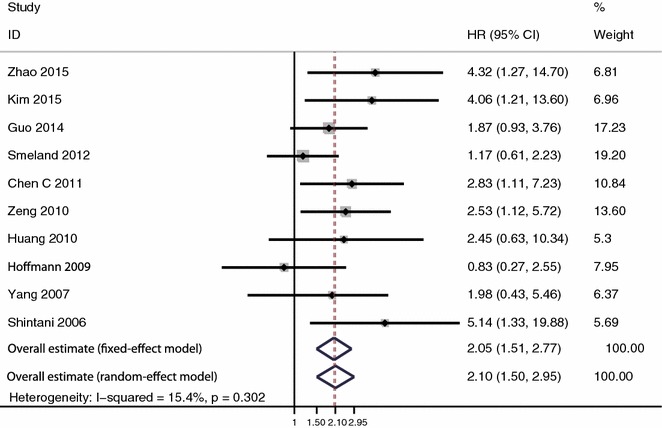
Meta-analysis of the effect of HIF-1α expression on overall survival

A total of 10 studies with 685 patients were included in the analysis of overall survival. The heterogeneity was not significant (I^2^ = 15.4 %, P = 0.302). Expression of HIF-1α was significantly associated with poor overall survival under fixed-effect model (HR 2.05, 95 % CI 1.51–2.77, P < 0.001) and random effect model (HR 2.10, 95 % CI 1.50–2.95, P < 0.001) (Fig. 2).

**Fig. 3:**
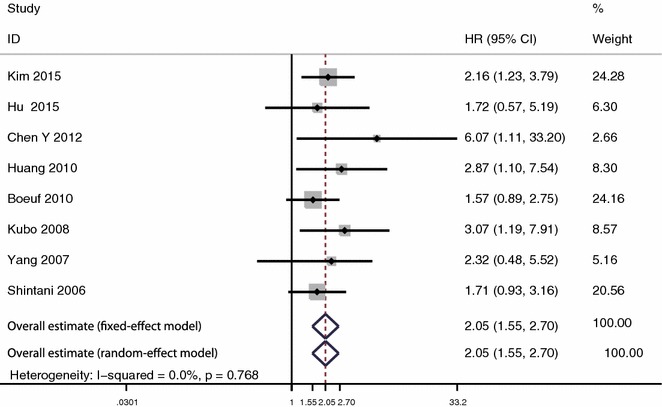
Meta-analysis of the effect of HIF-1α expression on disease-free survival

In the analysis of disease-free survival, 8 studies with 359 patients were included. The heterogeneity was not significant (I^2^ = 0.0 %, P = 0.768). The analysis indicated that expression of HIF-1α was significantly associated with poor disease-free survival under fixed effect model (HR 2.05, 95 % CI 1.55–2.70, P < 0.001) and random-effect model (HR 2.05, 95 % CI 1.55–2.70, P < 0.001) (Fig. 3).

**Fig. 4:**
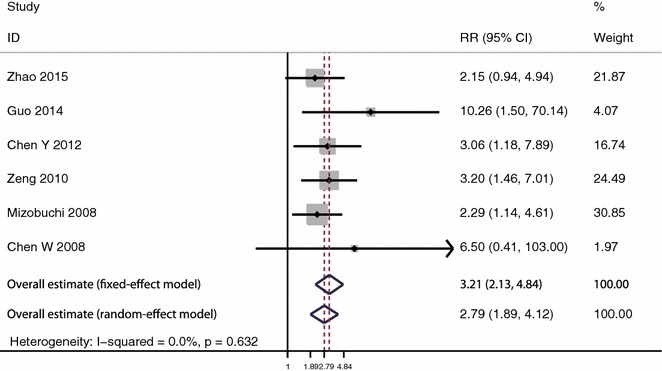
Meta-analysis of the effect of HIF-1α expression on metastasis

For metastasis, 6 studies with 363 patients were included. The heterogeneity was not significant (I^2^ = 0.0 %, P = 0.632). The analysis showed that HIF-1α expression was significantly associated with higher rate of metastasis under fixed effect model (RR 3.21, 95 % CI 2.13–4.84, P < 0.001) and random effect model (RR 2.79, 95 % CI 1.89–4.12, P < 0.001) (Fig. 4).

### Subgroup analysis of osteosarcoma

The pooled HR estimate for overall survival in osteosarcoma group was 2.32 (95 % CI 1.47–3.66) under both fixed-effect model and random-effect model. For disease-free survival, the pooled HR estimate was 2.43 (95 % CI 1.16–5.09) under both fixed-effect model and random-effect model. The heterogeneity was not significant (Table 2).

**Table 2:** Main results of meta-analysis

	No. of studies	Patients	Heterogeneity test (I^2^, P)	Combined estimate (95 % CI)/P value	
				Fixed-effect model	Random-effect model
Overall patients					
OS	10	685	15.4 %, 0.302	HR 2.05 (1.51–2.77)/<0.001	HR 2.10 (1.50–2.95)/<0.001
DFS	8	359	0.0 %, 0.768	HR 2.05 (1.55–2.70)/<0.001	HR 2.05 (1.55–2.70)/<0.001
MET	6	363	0.0 %, 0.632	RR 3.21 (2.13–4.84)/<0.001	RR 2.79 (1.89–4.12)/<0.001
Osteosarcoma					
OS	4	270	0.0 %, 0.692	HR 2.32 (1.47–3.66)/<0.001	HR 2.32 (1.47–3.66)/<0.001
DFS	3	138	0.0 %, 0.473	HR 2.43 (1.16–5.09)/0.018	HR 2.43 (1.16–5.09)/0.018
Soft tissue sarcoma					
OS	5	381	47.8 %, 0.105	HR 1.68 (1.07–2.63)/0.025	HR 1.94 (0.98–3.83)/0.055
DFS	3	136	0.0 %, 0.657	HR 2.06 (1.41–3.02)/<0.001	HR 2.06 (1.41–3.02)/<0.001
Chondrosarcoma					
OS	1	34	–	HR 2.83 (1.11–7.22)/0.030	HR 2.83 (1.11–7.22)/0.030
DFS	2	85	29.7 %, 0.233	HR 1.87 (1.15–3.04)/0.011	HR 1.96 (1.06–3.64)/0.033

*OS* overall survival, *DFS* disease-free survival, *MET* metastasis, *HR* hazard ratio, *RR* relative risk

### Subgroup analysis of soft tissue sarcoma

Under fixed-effect model, significantly poorer overall survival (HR 1.68, 95 % CI 1.07–2.63, P = 0.025) and disease-free survival (HR 2.06, 95 % CI 1.41–3.02, P < 0.001) were found in soft tissue sarcoma patients with expression of HIF-1α.However, under random effect model, the correlation to overall survival was not found to be significant (HR 1.94, 95 % CI 0.98–3.83, P = 0.055), while the correlation to poorer disease-free survival was still significant (HR 2.06, 95 % CI 1.41–3.02, P < 0.001).The heterogeneity was not found to be significant (Table 2).

### Subgroup analysis of chondrosarcoma

Significantly poorer overall survival (fixed-effect model: HR 2.83, 95 % CI 1.11–7.22; Random-effect model: HR 2.83, 95 % CI 1.11–7.22) and disease-free survival (fixed-effect model: HR 1.87, 95 % CI 1.15–3.04; Random-effect model: HR 1.96, 95 % CI 1.06–3.64) for HIF-1α expression were observed in chondrosarcoma patients without significant heterogeneity (Table 2).

### Sensitivity analysis

**Fig. 5:**
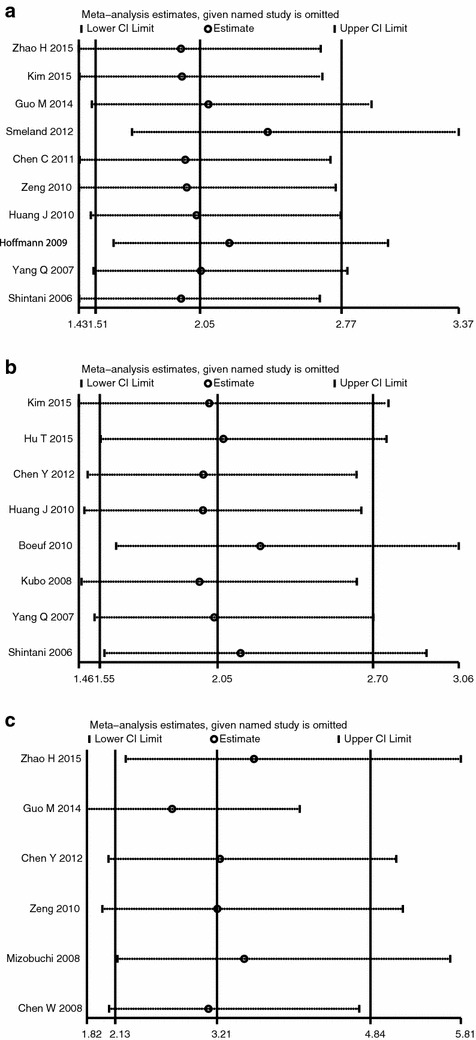
Sensitivity analysis of the effect of HIF-1α expression on **a** overall survival, **b** disease-free survival and **c** metastasis

By omitting one single study at a time, the effect of the study on the overall estimate could be investigated. The omitting of any study in the analyses of overall survival, disease-free survival and metastasis made no significant changes in the overall results, indicating that the analyses were statistically stable and reliable (Fig. 5).

### Evaluation of publication bias

**Fig. 6:**
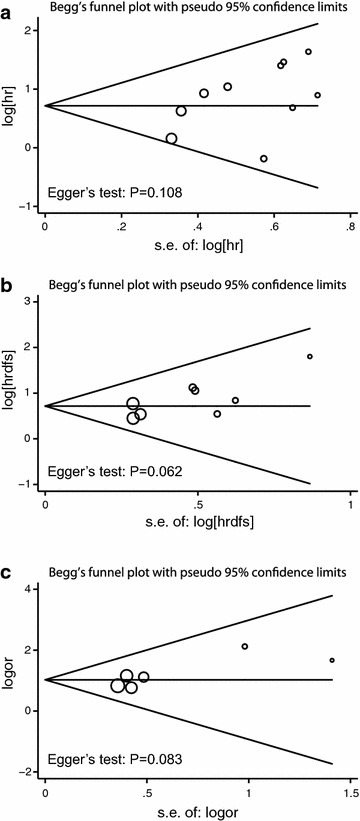
Begg’s funnel plot for publication bias of the correlation between HIF-1α expression and **a** overall survival, **b** disease-free survival and **c** metastasis

Egger’s test did not found any publication bias among the studies (P = 0.108, 0.062 and 0.083 for the analysis of overall survival, disease-free survival and metastasis, respectively). Visual evaluation of the Begg’s funnel plots found no apparent asymmetry (Fig. 6).

## Discussion

Bone and soft tissue sarcoma is the third leading cause of cancer related death in children and young adults (Siegel et al. 2015; Damron et al. 2007). With the emergence of effective chemotherapy regimens and the development of surgical techniques, the survival rate raised. However, metastasis is common and long-time survival of these patients is still poor (Nakamura et al. 2009; Tsukushi et al. 2014). Identification of effective prognostic factors is important to get a better understanding of the pathogenesis of bone and soft tissue sarcoma, and to develop new effective treatment methods. To date, several biomarkers have been discovered as prognostic factors of bone and soft tissue sarcoma, which play an important role in helping researchers and clinicians to choose ideal treatment methods (Zhuang and Wei 2014; Li and Geng 2010).

HIF-1α is an important regulator in cellular response to hypoxia in both malignant and normal tissues. Under hypoxia microenvironments, HIF-1α becomes stable and translocates from the cell plasma to the nucleus, where it dimerizes with HIF-1β and binds to the hypoxia response elements (HREs). Through the way, HIF-1α could regulate target genes which are associated with crucial aspects of tumor biology including angiogenesis, energy metabolism and vasomotor function, and thus make the tumor cell adaptive to the intratumoral hypoxia (Semenza 2001; Tsai and Wu 2012). Overall, the expression of HIF-1α contributes to the progression of many solid tumors through the way of sustaining energy metabolism, maintaining biosynthesis and promoting tumor cell invasion and migration (Stoeltzing et al. 2004). The prognostic significance of HIF-1α in tumors has been widely studied and literatures have identified that HIF-1α expression is an indicator for poor survival in several cancers (Zhang et al. 2013; Ping et al. 2014; Wang et al. 2014). However, its prognostic role in bone and soft tissue sarcoma has not been well established and reached a consensus. Therefore, we performed the meta-analysis to derive an overall pooled estimation of the association between HIF-1α expression and outcomes of sarcoma patients.

In the current meta-analysis, we combined 16 studies with 942 sarcoma patients comparing the outcomes of metastasis and survival according to the level of HIF-1α expression. We found that expression of HIF-1α was significantly associated with poor overall survival, poor disease-free survival and higher rate of metastasis. In addition, when subgroup analysis was conducted according to histology type, the significant correlations to poor overall survival and disease-free survival were also observed in patients with osteosarcoma, chondrosarcoma and soft tissue sarcoma. The sensitivity analysis showed that the results were statistically stable and reliable. Therefore, HIF-1α may be an effective prognostic factor of poor prognosis for bone and soft tissue sarcoma. To our knowledge, it is the first time to systematically evaluate the prognostic role of HIF-1α expression in bone and soft tissue sarcoma.

As the hazard ratio was chose to assess the prognostic significance, it is important to also mention the changes in the time-to-event measurement. In majority of the included studies, the disease-free and overall survival rate of patients with positive expression of HIF-1α was lower than those with negative expression from the beginning of follow-up. The gap between the two groups would be enlarged as the follow-up duration went on. In particular, only one of the included studies did not show this time-to-event pattern (Smeland et al. 2012), in which the survival curves of the two groups were continuous intersected, with a slightly trend of favorable survival for the negative expression group.

The heterogeneity was not found to be significant in the analyses, however, it should be noted that our meta-analysis could not totally excluded biases, which could be arisen from several aspects. Firstly, the methods of quantitative HIF-1α measurement differed among these studies. Although the most common method was IHC, the studies did not use the same antibody, and its dilutions were also different. Because the type and the concentration of the antibody could affect the sensitivity of IHC, the differences may lead to a potential bias. In addition, differences also existed in the cut-off value to determine the positive expression of HIF-1α. To date, there have been no uniform criteria for the methodology and determination of HIF-1α expression using IHC method. Thus, these methodological variances could bring heterogeneity and lower the reliability of pooled results. However, because of the small groups of studies using the same antibody and cut-off value, we could not perform a subgroup analysis to clarify this technical problem, and uniform criteria are urgently needed for future studies to draw a more homogeneous conclusion.

To ensure the accuracy of the pooled results and minimize its bias derived from the heterogeneity among the included studies, we calculated the HRs or RRs with both fixed and random effect models adopted. In majority of the analyses, the pooled results under fixed and random effect models were consistent (Table 2). Only in the analysis of overall survival in patients with soft tissue sarcoma, the result under random effect model was negative (P = 0.055), which was inconsistent with the result under fixed effect model (P = 0.025). Nevertheless, an obvious tendency could be observed in the negative result (HR 1.94, 95 % CI 0.98–3.83). Thus, it is reasonable to consider that the pooled results of the current meta-analysis were relatively accurate and the bias was limited.

It is also worthy to mention the method to extrapolate HRs or RRs from the included articles. When these data from multivariate survival analysis were reported, we used them directly. If the HRs or RRs were not given explicitly, we calculated them from outcome data available in the articles. If this was impracticable, we extrapolated them from Kaplan–Meier curves by univariate analysis (Xu et al. 2013; Zhuang and Wei 2014; Kubo et al. 2015). The estimation might be less reliable than the HRs given directly in the papers. Therefore, the results of the current meta-analysis should be interpreted with caution and should be confirmed by more well-designed prospective studies with appropriate multivariate analyses.

Publication bias is another major concern in all forms of meta-analyses, since positive results trend to be published in journals. To minimize publication bias, we attempted to perform literature searches as complete as possible, using Web of Science, PubMed, Embase and Cochrane Library. The publication bias was not found in our analyses, however, it should be noted that we only included articles in English or Chinese. Secondly, we excluded conference abstracts since it did not contain sufficient information for aggregation. Besides, the Begg’s test has relative low power to detect the publication bias if the number of included studies were not large (Sterne et al. 2000), and P values of the Egger’s test are nearing 0.05 (Fig. 6). Thus, these restrictions may bring potential source of publication bias to the current meta-analysis.

In conclusion, this meta-analysis demonstrates that HIF-1α expression may be an effective predicative factor of poor prognosis for bone and soft tissue sarcoma. Further well-designed prospective studies are needed to validate our findings.
